# Disruption of *CmHmgr1* triggers apoptosis and causes defects in growth, conidiogenesis, and mycoparasitism of *Coniothyrium minitans*

**DOI:** 10.1080/21505594.2025.2523884

**Published:** 2025-07-23

**Authors:** Xiaoxiang Yang, Haixuan Wang, Lei Zhang, Zhongmei Zhang, Zijin Hu, Daohong Jiang, Yanping Fu

**Affiliations:** a Institute of Plant Protection, Sichuan Academy of Agricultural Sciences, Chengdu, China; b Key Laboratory of Integrated Pest Management on Crops in Southwest, Ministry of Agriculture and Rural Affairs, Chengdu, China; c The Provincial Key Lab of Plant Pathology of Hubei Province, College of Plant Science and Technology, Huazhong Agricultural University, Wuhan, Hubei, China; d National Key Laboratory of Agricultural Microbiology, Huazhong Agricultural University, Wuhan, Hubei, China

**Keywords:** *Coniothyrium minitans*, HMGR, apoptosis, conidiation, mycoparasitism, *Sclerotinia sclerotiorum*

## Abstract

*Coniothyrium minitans* is a well-known mycoparasite against *Sclerotinia sclerotiorum.* Two critical factors for the commercialization of *C. minitans* as a biocontrol agent are conidial production and parasitism. To decipher the mechanisms of conidiogenesis and mycoparasitism in *C. minitans*, a conidiation-deficient mutant, ZS-1TN5012, was isolated from a transfer DNA (T-DNA) insertional library. This mutant exhibited significantly reduced hyphal development, poor conidiation, and decreased sclerotial mycoparasitism. *CmHmgr1* encoding a 3-hydroxy-3-methylglutaryl coenzyme A reductase (HMGR) was disrupted by the T-DNA insertion. The colony morphology of the wild-type strain ZS-1 resembled that of the mutant ZS-1TN5012 when the HMGR inhibitor atorvastatin was added to potato dextrose agar, with the mutant showing more sensitivity to atorvastatin. Furthermore, cellular localization assays revealed that CmHmgr1 was localized in mitochondria. Gene replacement and complementation experiments confirmed that *CmHmgr1* is involved in the growth, conidiogenesis and mycoparasitism of *C. minitans*, and disruption of *CmHmgr1* triggers apoptosis.

## Introduction

The fungus *Sclerotinia sclerotiorum* is a widespread plant pathogen responsible for *Sclerotinia* diseases in numerous plant species, including oilseed rape, lettuce, carrot, vegetable brassicas, peas, and beans, resulting in significant economic losses annually [1,2]. *Coniothyrium minitans*, an important sclerotial and hyphal parasite of *S. sclerotiorum*, is recognized for its potential in controlling crop diseases caused by *S. sclerotiorum*, *S. minor*, *S. cepivorum*, and *S. trifoliorum* [3]. For its commercialization as a biological control agent, conidial production and parasitic ability are critical considerations [4]. Therefore, understanding of the mechanisms of conidiation and mycoparasitism in *C. minitans* is essential for optimizing its use as a biological control agent and enhancing our knowledge of fungal biology.

A library of T-DNA insertion mutants of *C. minitans* was constructed using ATMT technology [5], and the analysis of the identified genes showed that the conidiogenesis and parasitism of *C. minitans* were likely very complicated and related to several genes and pathways, including signaling mediated by cyclic GMP (cGMP), cyclic AMP (cAMP), nitric oxide, the mitogen-activated protein (MAP) kinase (MAPK) cascade, peroxisome biogenesis, the NADPH oxidase complex, autophagy, and altered inheritance of mitochondria protein, etc. [6–13]. It is still necessary to excavate conidiogenesis or parasitism-related genes, discover new pathways, explore the crossover of pathways, and map the regulatory network of conidiogenesis and parasitism of *C. minitans*.

The endoplasmic reticulum (ER)-localized enzyme 3-hydroxy-3-methyl-glutaryl CoA reductase (HMGR) plays a crucial role in the mevalonate pathway. This pathway controls a rate-limiting step in the conversion of HMG-CoA into mevalonate, a precursor for various isoprene-containing compounds, including sterols, dolicol, ubiquinone, and isopentenylated tRNAs [14,15]. These end products have diverse cellular roles, such as maintaining membrane structure, electron transport, glycoprotein biosynthesis, translation, and DNA replication [16]. As a key enzyme in a pathway that produces compounds with significant functions in eukaryotes, HMGRs have been characterized on its regulatory mechanisms in secondary metabolisms, as well as the development of medicinal plant resources and the treatment of cardiovascular diseases [17–19]. However, researches on the impact of HMGR on the growth and development are relatively weak. In *Arabidopsis thaliana*, two genes encode HMGR: *hmg1* and *hmg2*. The *hmg2* mutant shows no abnormal phenotype, whereas the *hmg1* mutant shows pleiotropic phenotypes, including dwarfism, early senescence, and male sterility [20]. Similarly, in *Saccharomyces cerevisiae*, there are two genes encoding HMGR: *HMG1* and *HMG2*. *HMG1* or *HMG2* mutants show defects in spore germination and vegetative growth and are more sensitive to the inhibitors of HMGR than the wild-type strain. However, cells bearing null alleles of both *HMG1* and *HMG2* are inviable [21]. Currently, it remains unclear whether HMGR regulates the growth, conidiogenesis, and mycoparasitism of filamentous fungi.

To decipher the mechanisms of conidiogenesis and mycoparasitism in *C. minitans*, we previously constructed a transfer DNA (T-DNA) insertional library of the *C. minitans* strain ZS-1 [5]. From this mutant library, a conidiation-deficient mutant, ZS-1TN5012, was screened. *CmHmgr1* encoding HMGR was disrupted by a T-DNA insertion in ZS-1TN5012. In this study, roles of *CmHmgr1* in hyphal growth, conidiogenesis, mycoparasitism, and apoptotic processes in *C. minitans* were explored.

## Experimental procedures

### Strains and culture conditions

The *C. minitans* wild-type strain ZS-1 (CCAM041057), which regularly produces pycnidia and conidia [22], was isolated from the garden soil at Zhushan County, Hubei Province, PR China. The conidiation-deficient mutant ZS-1TN5012 was screened from a T-DNA insertional library of ZS-1 [5]. Strain Ep-1PNA367 is a virulent strain of *Sclerotinia sclerotiorum* [23]. All strains were cultured on potato dextrose agar (PDA) at 20°C and stored in PDA slants at 4°C. For the assessment of tolerance to atorvastatin (Sigma), final atorvastatin concentrations of 5 μg/mL and 20 μg/mL were used.

### Phenotypic analysis

Hyphal agar plugs (diameter 5 mm) were taken at the edge of the colony activated on PDA plates for 3–4 days and transferred to the center of fresh 20 mL PDA plates (diameter 90 mm). The growth rate, conidial production, and conidial germination were examined as described previously [12,24]. Observation of sclerotial parasitization of *C. minitans* was performed according to Zeng et al. [13] was used to assess the extent of parasitic activity of *C. minitans*.

### Analysis of the gene disrupted by T-DNA insertion

The mutant ZS-1TN5012 and the wild-type strain ZS-1 were cultured on PDA, and mycelia were harvested after 4 days of incubation at 20°C. Genomic DNA of ZS-1TN5012 and ZS-1 were extracted using the cetyltrimethylammonium bromide (CTAB) method. To obtain the sequences flanking the T-DNA insertion site, the hiTAIL-PCR amplification was used following the method described by Yang et al. [12]. Southern blot analysis was used to check the copy of T-DNA in ZS-1TN5012, following the method described by Gong et al. [6].

### Vector construction and agrobacterium-mediated transformation

Targeted deletion of *CmHmgr1* was performed using the split marker technique [25,26]. Two overlapping gene deletion constructs were generated following the method described by Yang et al. [12]. Primers UpHmgrF, UpHmgrR, HYG-F, HY-R, DpHmgrF, DpHmgrR, YG-F, and HYG-R are shown in Table S1. These two overlapping DNA constructs were used to transform protoplasts of strain ZS-1.

For complementation of the *CmHmgr1* mutant, the full-length *CmHmgr1* gene coding region and a 1287 bp downstream sequence were amplified using the primer pair CHmgrF and CHmgrR and cloned into neoP3300 to generate the complementary vector pCHmgr. pCHmgr was subsequently transformed into the ΔCmHmgr-1 mutant using the *Agrobacterium tumefaciens*-mediated transformation (ATMT) method as previously described [9,13].

For cellular localization of CmHmgr1 in *C. minitans*, a red fluorescent protein (RFP) fragment amplified with primers RFPF and RFPR was fused to the 5´-end of the *CmHmgr1* gene and used to generate the pCmHmgr1-RFP vector. pCETNS-RFP with RFP fragment only was used as a control. pCmHmgr1-RFP and pCETNS-RFP were then transformed into the ΔCmHmgr-1 using the ATMT method. Mycelia were cultured at 20°C for 3 days and stained with the membrane potential specific dye MitoTracker Green (Beyotime) following the manufacturer’s instructions. The stained mycelia were observed using a laser confocal microscope (OLYMPUS® microscope FV1000).

### RNA extraction and qRT-PCR analysis

The total RNA of mycelia at 72 hpi was isolated, and first-strand cDNA synthesis was performed according to the method described by Yang et al. [11]. In quantitative real-time PCR (qRT-PCR), *CmHmgr1* (primer pair CmHmgr1-F/CmHmgr1-R), *CmYca1* (primer pair CmYca1-F/CmYca1-R), *CmAif1* (primer pair CmAif1-F/CmAif1-R), *CmNdi1* (primer pair CmNdi1-F/CmNdi1-R), *CmNma111* (primer pair CmNma111-F/CmNma111-R) and *CmBix1* (primer pair CmBix1-F/CmBix1-R) were amplified using the respective primer pairs (Table S1). As an endogenous control, a 154-bp amplicon of *actin* was amplified with primer pair Actin-F and Actin-R (Table S1). The relative expression level was calculated by the 2^−ΔΔCt^ method.

### Annexin V-FITC/PI staining and TUNEL assay

For Annexin V-FITC/PI-staining, mycelia were digested with lyticase for 20 min following Franco et al. [27] and stained using the Annexin V-FITC/PI Apoptosis Detection Kit (MeilunBio) according to the manufacturer’s instructions. For terminal deoxynucleotidyl transferase-mediated dUTP nick end labeling (TUNEL), mycelia were fixed with 4.0% formaldehyde for 30 min followed by digestion of the cell walls with lyticase 30 min [28]. Then, the cell permeabilization and TUNEL reactions were carried out using a TUNEL Apoptosis Detection Kit (YSFluor^TM^640) (Yeasen) according to the manufacturer’s instructions. The stained mycelia were observed using a laser confocal microscope (OLYMPUS® microscope FV1000).

### Data analyses

Data from the experiments were analyzed using an analysis of variance (ANOVA) performed on SAS version 8.1 (SAS Institute, Inc., Cary, NC). When significant treatment differences were found, treatment means were separated using the protected least significant difference (LSD) test at *P*=0.05.

## Results

### Characterization of conidiation-deficient mutant ZS-1TN5012 of *C. minitans*

**Figure 1.**
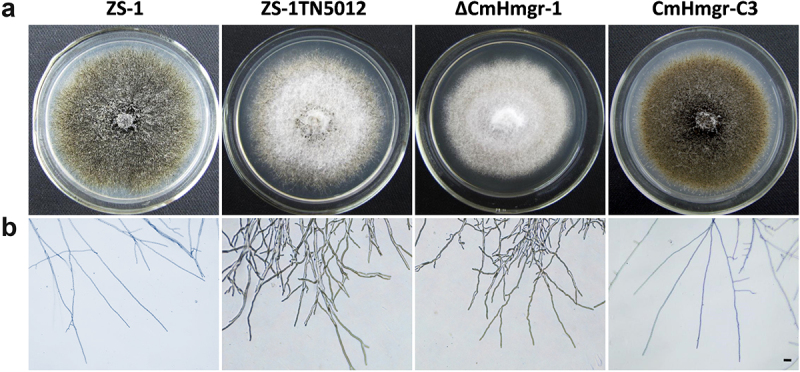
Comparison of colony morphology and branching pattern among ZS-1, ZS-1TN5012, ΔCmHmgr-1, and CmHmgr-C3. (a) Colony morphology of *C. minitan*s. Images were taken 10 days after incubation on PDA at 20°C. (b) Branching pattern was observed 3 days after incubation on PDA at 20°C, scale bar = 10 μm.

The ZS-1TN5012 mutant formed a light-colored colony with fewer dark pycnidia and showed significantly reduced hyphal growth. In contrast, the wild-type strain ZS-1 formed dark-colored colonies with abundant mature pycnidia (Figure 1a). The conidial production by ZS-1TN5012 (1.3 × 10^6^ conidia per cm^2^) was approximately 80-fold less than that of the wild-type strain ZS-1 (99 × 10^6^ conidia per cm^2^) at 15 days post incubation (Table 1). Additionally, the diameter of the hyphal tips at the colony margin of ZS-1TN5012 was larger with more branches compared to those of strain ZS-1 (Figure 1b).

**Table 1.** Comparison of hyphal growth, conidial production, and germination of *C. minitans.*

Strains	Growth rate (mm/d)	Conidiation (×10^6^conidia/cm^2^)	Conidia germination (%)
ZS-1	3.09 ± 0.07^a^	99.96 ± 0.57^a^	89.58 ± 1.55^a^
ZS-1TN5012	2.50 ± 0.16^b^	1.3 ± 0.07^b^	31.08 ± 2.61^b^
ΔCmHmgr-1	2.35 ± 0.09^b^	0.7 ± 0.02^b^	30.14 ± 0.48^b^
CmHmgr-C3	3.14 ± 0.25^a^	101.02 ± 0.38^a^	91.16 ± 4.06^a^

” >Growth rate was detected by measuring the colony diameter of cultures incubated at 20°C for 10 days. Conidia produced 15 days post-incubation were counted using a hemocytometer. Conidial germination of all strains was measured at 36 hpi. Means and standard errors were calculated from three replicates.

### Cloning and analysis of *CmHmgr1*

**Figure 2.**
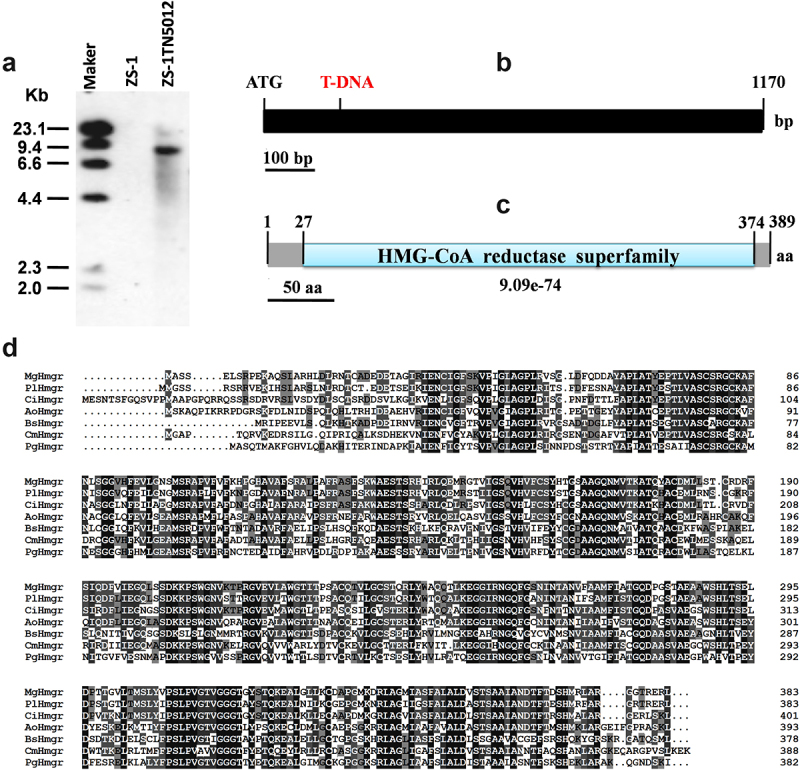
Analysis of the T-DNA insertional events in mutant ZS-1TN5012. (a) Southern blot analysis of the copy number of T-DNA in ZS-1TN5012. (b) The T-DNA insertion site in *CmHmgr1*, the black segment indicates the exon. (c) Analysis of putative conserved domains encoded by *CmHmgr1*. The 9.09e-74 is the E-value of protein domain similarity. (d) Alignment analysis of protein CmHmgr with MgHmgr (KID85852), PlHmgr (OAQ60393), CiHmgr (KZL84138), AoHmgr (XP_001825169), BsHmgr (GAD93336), and PgHmgr (KXG45640).

Only one copy of T-DNA was inserted into the genome of the mutant ZS-1TN5012 as shown by Southern blot analysis (Figure 2a). Then, hiTAIL-PCR technique was used to amplify the T-DNA flanking genomic DNA sequence in ZS-1TN5012. The obtained flanking sequence contained the left border of the inserted T-DNA and an incomplete open reading frame (ORF). The coding region was identified by comparing it to the *C. minitans* genome. *CmHmgr1*, which encodes HMGR, was found disrupted by T-DNA insertion (Figure 2c).

*CmHmgr1* (GenBank Acc. No. KY949479) is predicted to consist of 1170 bp and encodes a protein of 389 amino acids. In the ZS-1TN5012 mutant, *CmHmgr1* was disrupted by T-DNA insertion at the nucleotide position 166 nt after the translational start codon (ATG) (Figure 2b). The deduced amino acid sequence of *CmHmgr1* shares similarity with HMGR homologs from various fungi (Figure 2d).

### The effects of atorvastatin on *C. minitans* hyphal growth

**Figure 3.**
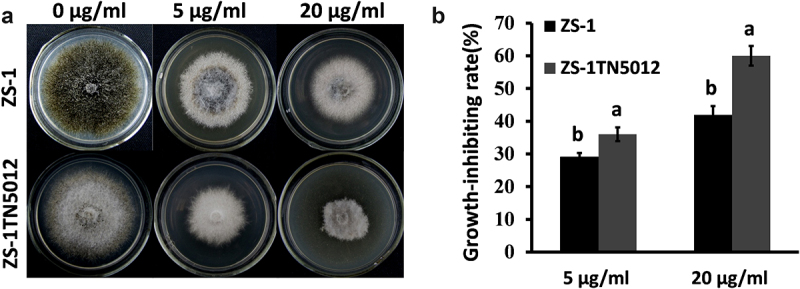
Assessment of the tolerance to atorvastatin in *C. minitans* strains. (a) The colony morphology and (b) growth-inhibiting rate of all strains on PDA media supplemented with atorvastatin. Error bars represent the standard deviations, and columns with the same letters are not significantly different (*P*>0.05) according to a least significant difference test.

Statins are well-known inhibitors of HMGR. In this research, the effects of atorvastatin on the hyphal growth of *C. minitans* were evaluated. Both the wild-type strain ZS-1 and ZS-1TN5012 exhibited lighter pigmentation and significantly reduced hyphal development when incubated on PDA added with atorvastatin (Figure 3a). Moreover, ZS-1TN5012 was more sensitive than strain ZS-1 (Figure 3b).

### Targeted disruption and complementary of *CmHmgr1*

**Figure 4.**
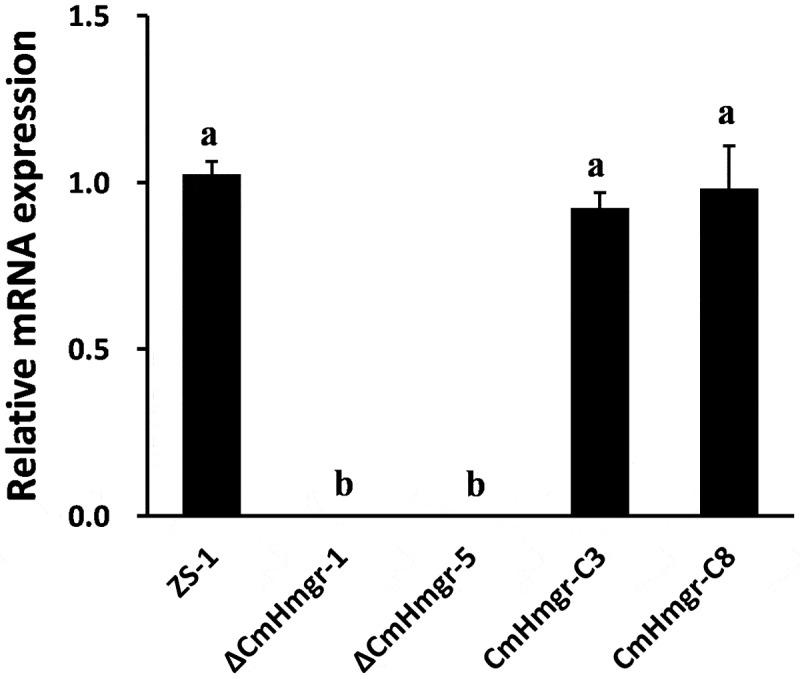
Confirmation of targeted disruption and complementary strains by qRT-PCR. RNA samples at 72 hpi were used to examine the relative expression levels of *CmHmgr1*. Error bars represent the standard deviations, and columns with the same letters are not significantly different (*P*>0.05) according to a least significant difference test.

To determine the role of *CmHmgr1* in the conidiogenesis and hyphal growth of *C. minitans*, targeted gene replacement was performed using the split marker technique. Two deletion mutants (ΔCmHmgr-1 and ΔCmHmgr-5), confirmed by qRT-PCR (Figure 4), were selected for further analysis. For complementation of the *CmHmgr1* mutant, the complementary vector pCHmgr1 was transformed into ΔCmHmgr-1, and two complemented strains (CmHmgr-C3 and CmHmgr-C8), also confirmed by qRT-PCR, were selected for further analysis (Figure 4).

### *CmHmgr1* affects growth, conidiogenesis, and conidial germination

Like the T-DNA insertional mutant ZS-1TN5012, ΔCmHmgr-1 exhibited lighter color and more branched hyphae (Figure 1). The growth of ΔCmHmgr-1 was reduced by approximately 25% compared to the wild-type ZS-1 on PDA (Table 1). The conidial production of ΔCmHmgr-1 was approximately 100-fold less than that of the wild-type strain ZS-1 (Table 1). Moreover, conidial germination of the ΔCmHmgr-1 was significantly inhibited, with a germination rate of about 30% for mutants at 36 hpi. In contrast, the conidia germination rate of ZS-1 and CmHmgr-C3 was 90% (Table 1). These results indicate that *CmHmgr1* is required for growth, conidiogenesis, and conidial germination.

### *CmHmgr1* is involved in mycoparasitism

**Figure 5.**
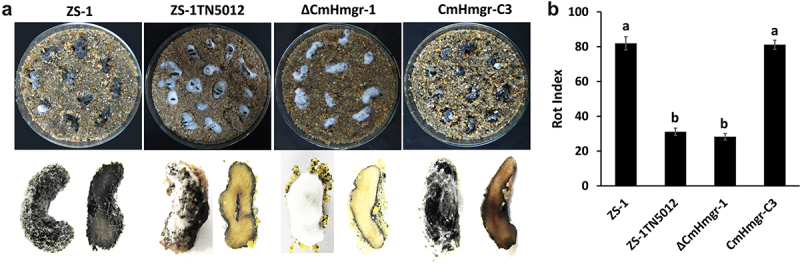
Parasitic activity assays of *C. minitans* to sclerotia of *S. sclerotiorum*. (a) Observation of sclerotial parasitization of *C. minitans*. Sclerotia treated with sterile water were used as control. (b) Rot index of all the strains. Error bars represent the standard deviations, and columns with the same letters are not significantly different (*P*>0.05) according to a least significant difference test.

The ability to parasitize *S. sclerotiorum* sclerotia is crucial for the commercialization of *C. minitans*. The parasitic activity of ZS-1TN5012 and ΔCmHmgr-1 was dramatically reduced. These mutants were able to colonize sclerotia but produced fewer dark pycnidia on the surface of *S. sclerotiorum* even after 30 days of incubation, and intact pith was observed inside the bisected sclerotia. In contrast, the wild-type strain and complementary strain produced abundant mature pycnidia on the surface of the sclerotia, and the infected sclerotia showed degraded pith (Figure 5a). The rot index of ZS-1TN5012 and ΔCmHmgr-1 was reduced by more than 50% compared to strains ZS-1 and CmHmgr-C3 after 30 days of incubation (Figure 5b). These results suggest that *CmHmgr1* is required for the mycoparasitism of *C. minitans*.

### Subcellular localization of CmHmgr1

**Figure 6.**
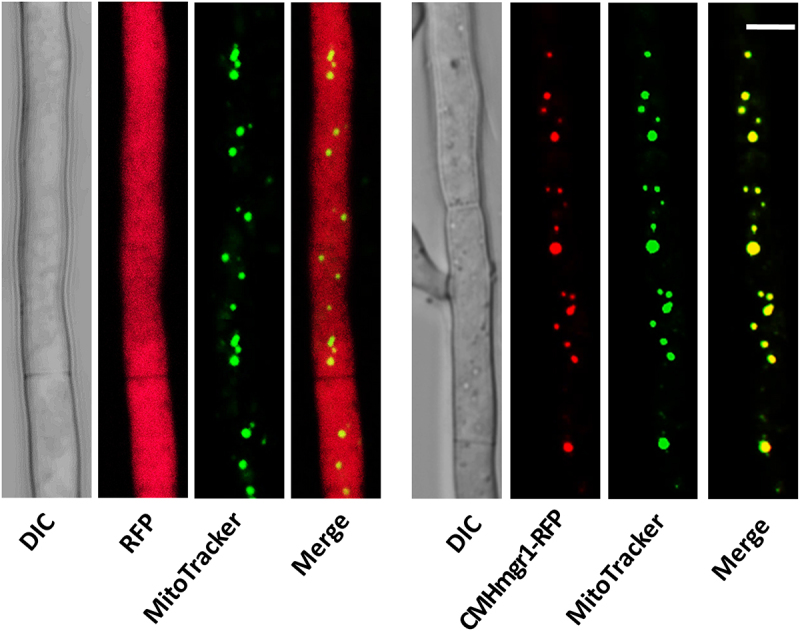
Subcellular localization of CmHmgr1. A laser confocal micrograph shows the colocalization of CmHmgr1 with mitochondria stained with the membrane potential-specific dye MitoTracker in the ΔCmHmgr-1 mutant transformed with the CmHmgr1-RFP expression cassette. The RFP signals were uniformly distributed in hyphae of the ΔCmHmgr-1 mutant transformed with RFP only. DIC, differential interference contrast. Scale bar = 2 μm.

CmHmgr1 is most likely to be located in mitochondria predicted by WoLF PSORT. To confirm the subcellular localization of CmHmgr1, a CmHmgr1-RFP fusion expression cassette was constructed and transformed into ΔCmHmgr-1. The defects in ΔCmHmgr-1 were complemented by expressions of CmHmgr1-RFP, indicating that this fusion protein was functional. RFP signals were found exclusively in mitochondria. When mitochondria were stained with the membrane potential specific dye MitoTracker Green, a clear colocalization of CmHmgr1 with mitochondria was detected (Figure 6). In contrast, as a control, the RFP signals were uniformly distributed in hyphae of ΔCmHmgr-1 transformed with only RFP (Figure 6). These observations suggest that CmHmgr1 is localized in mitochondria of *C. minitans*.

### Disruption of *CmHmgr1* induces apoptosis

**Figure 7.**
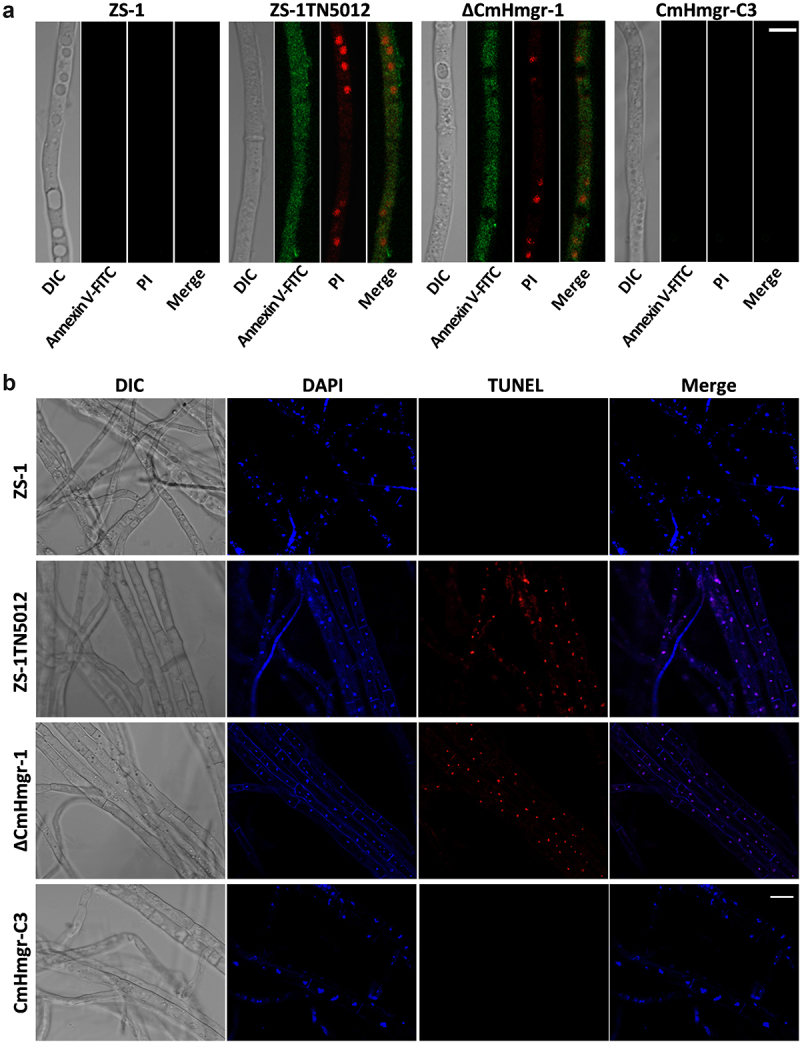
Detection of apoptosis of *C. minitans* using confocal laser scanning microscopy. (a) Annexin-FITC/PI double staining to evaluate apoptosis, scale bar = 2 μm. (b) TUNEL assay of *C. minitans* strains, fungal cells were also stained with DAPI to visualize nuclei, scale bar = 10 μm. DIC, differential interference contrast.

Annexin-FITC/PI double staining and TUNEL assay were used to evaluate the apoptosis. FITC-labeled Annexin V binds specifically to phospholipids (PS) flipped out to the outer membrane during apoptosis and emits green fluorescence. PI cannot penetrate through normal cell membranes but can pass through necrotic cell membranes and bind to DNA in the nucleus and emit red fluorescence. Both green and red fluorescence were only detected in ZS-1TN5012 and ΔCmHmgr-1 (Figure 7a). In TUNEL assay, clear TUNEL signals were detected in the mycelial nuclei of ZS-1TN5012 and ΔCmHmgr-1, but not in ZS-1 and complemented strain CmHmgr-C3 (Figure 7b). The results indicate that the apoptosis was triggered by the disruption of *CmHmgr1*.

**Figure 8.**
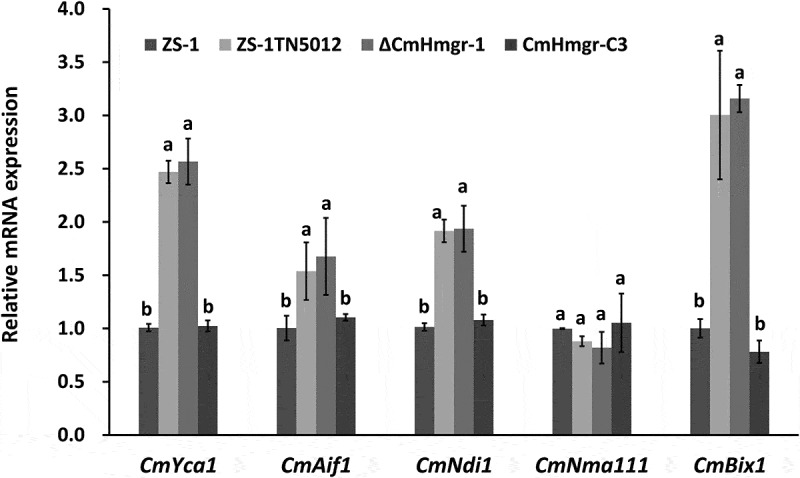
Relative expression levels of apoptosis-related genes. Error bars represent the standard deviations, and columns with the same letters are not significantly different (*P*>0.05) according to a least significant difference test.

The expressions of apoptosis-related genes were further examined by qRT-PCR. The expression of *CmYca1*, *CmAif1,* and *CmNdi1* were significantly up-regulated in the mutants. Surprisingly, the anti-apoptotic gene *CmBix* (a *Bcl-2*-like gene) was also up-regulated in the mutants (Figure 8), possibly because the occurrence of apoptosis in the mycelia triggered an anti-apoptotic program to maintain cell survival.

## Discussion

It has been reported that conidiogenesis and mycoparasitism of *C. minitans* can be regulated by cGMP, cAMP, nitric oxide, MAPK cascade, peroxisome biogenesis, autophagy, etc. In this study, we revealed a new conidiogenesis and parasitism-related gene, *CmHmgr1*, and its possible mechanism. This study provided theoretical basis for construction and optimization of engineering strains and promotion of large-scale industrialization and application of biocontrol agents of *C. minitans.*

The ER-localized HMGR is a rate-limiting enzyme in mevalonate and isoprene-containing compounds biosynthesis. Isoprene-containing compounds are crucial for the survival of both eukaryotic and prokaryotic cells, as they participate in a diverse range of processes [29]. For instance, ergosterol, an important constituent of fungal membrane lipids, is essential for cell membrane fluidity, permeability, and pheromone signaling [30,31]. Research shows that the ergosterol biosynthesis pathway affects the development of fungi. Deletion of the transcription factor Sre1 which regulates the ergosterol biosynthesis in *Cryptococcus neoformans*, caused deficient in sexual development [30]. Deletion of FgErg4, FgErg3, or FgErg5—the enzymes participate in ergosterol synthesis in *Fusarium graminearum*—results in significant decreases in hyphal growth, conidiation, and pathogenicity [32,33]. In this study, we found CmHmgr1 is localized in mitochondria and the defects of ΔCmHmgr-1 cannot be rescued by exogenous mevalonate or ergosterol (Figure S1), possibly due to insufficient exogenous ergosterol or mevalonate uptake by *C. minitans*, similar to *F. graminearum* [32] and *S. cerevisiae* [21], or the function of mitochondria-localized HMGR is different from ER-localized HMGR.

Apoptosis is a process of programmed cell death and plays an important role in fungal development. The apoptosis-associated gene *MoFIS1* regulates fungal growth, conidiation, and virulence in *Magnaporthe oryzae*; *MoFIS1* deletion mutants are severely defective in colony growth, conidiation, virulence on rice and barley, as well as growth under nitrogen and glucose deficiency and osmotic stress [34,35]. The deletion of apoptosis-associated gene *FpBIR1* or *FpNUC1* also exhibits defects in conidiation, conidial germination, and pathogenicity of *F. pseudograminearum* [36]. The mitochondrion is considered a regulatory center of apoptosis [37]. CmHmgr1 is localized in mitochondria, and disruption of *CmHmgr1* triggers apoptosis and causes defects in growth, conidiogenesis, and pathogenicity to *S. sclerotiorum*. Moreover, the expressions of apoptosis-related genes were significantly up-regulated in the mutants. Therefore, we hypothesized that *CmHmgr1* affected growth, conidiogenesis, and mycoparasitism of *C. minitans*, probably also by regulating the apoptosis pathway.

Examination of the genome revealed two HMGR encoding genes, *CmHmgr1* and *CmHmgr2*, were present in *C. minitans*. *CmHmgr1* consists of 1170 bp and encodes a protein of 389 amino acids which is localized in mitochondria, while *CmHmgr2* consists of 3646 bp and encodes a protein of 1124 amino acids which is predicted to be located in the ER membrane. The transcriptome data (SRR9988701 to SRR9988715, https://www.ncbi.nlm.nih.gov/) of interaction between *C. minitans* and *S. sclerotiorum* were used to analyze the expression dynamics of *CmHmgr1* and *CmHmgr2*. It was found that *CmHmgr1* was induced during the interaction with *S. sclerotiorum*, while the expression of *CmHmgr2* was not affected (Figure S2). It is further indicated that *CmHmgr1* plays an important role in mycoparasitism of *C. minitans*, and *CmHmgr2* may have different functions. Unfortunately, the *CmHmgr2* deletion mutants were not obtained despite several attempts, thus the effect function of *CmHmgr2* is unknown.

## Conclusions

Disruption of *CmHmgr1*, encoding a mitochondria-localized 3-hydroxy-3-methylglutaryl coenzyme A reductase (HMGR), triggers apoptosis and causes defects in growth, conidiogenesis, and mycoparasitism of *Coniothyrium minitans*, a mycopersite of against *Sclerotinia sclerotiorum*.

## Supplementary Material

Table S1.docx

Figure S1.tif

Figure S2.tif

## Data Availability

Raw data are available via Figshare (https://doi.org/10.6084/m9.figshare.28031288.v2). The transcriptomics data for the expression dynamics of *CmHmgr1* and *CmHmgr2* during the interaction of *C. minitans* and *S. sclerotiorum* have been deposited in the Sequence Read Archive (SRA) on NCBI (https://www.ncbi.nlm.nih.gov/) under accessions of SRR9988701 to SRR9988715.
