# Supplementary material for: Disruption of CmHmgr1 triggers apoptosis and causes defects in growth, conidiogenesis, and mycoparasitism of Coniothyrium minitans
Source: Virulence. 2025 Jul 23;16(1):2523884. doi: 10.1080/21505594.2025.2523884 (PMC12296070; doi:10.1080/21505594.2025.2523884)
Supplement: Table S1.docx [file KVIR_A_2523884_SM9339.docx]

**Table S1 primers used in this study**

| **Primers** | **Sequence (5′-3′)** |
| --- | --- |
| 5012F | TCGCTCCATCCTCGGTCAAAT |
| 5012R | CGGCATCCCCACAAGAGTAG |
| XLB1 | TTTCTCCATAATAATGTGTGAGTAGTTCCCAGAT |
| XLB2 | ACGATGGACTCCAGTCCGGCCGGGTTTCGCTCATGTGTTGAGCATATAAG |
| XLB3 | CGTTAATTCAGTACATTAAAAACGTCCGCAAT |
| RB-0a | GGCAATAAAGTTTCTTAAGATTGAATCCTGT |
| RB-1a | ACGATGGACTCCAGTCCGGCCTGTTGCCGGTCTTGCGATGATTATCA |
| RB-2a | GTAATGCATGACGTTATTTATGAGATGGGTT |
| LAD1-1 | ACGATGGACTCCAGA GCGGCCGC(G/C/A) N(G/C/A)NNNGGAA |
| LAD1-2 | ACG ATG GACTCCAGA GCGGCCGC(G/C/T)N(G/C/T)NNNGGTT |
| LAD1-3 | ACGATGGACTCCAGAGCGGCCGC(G/C/A)(G/C/A)N(G/C/A)NNNCCAA |
| LAD1-4 | ACGATGGACTCCAGAGCGGCCGC(G/C/T)(G/A/T)N(G/C/T)NNNCGGT |
| AC1 | ACGATGGACTCCAGAG |
| UpHmgrF | CCGTTGATTCTTAGTGAGGTGATGC |
| UpHmgrR | CGAGGGCAAAGGAATAGAGTAGATGCCGAAGGTGATTACGCTGAAAGAGGGTG |
| DpHmgrF | GCTCCTTCAATATCATCTTCTGTCGACTCTAGAGCTTTGCCCTCGTCATTGC |
| DpHmgrR | GTCGCCGCCTAATCTCTTACTACAG |
| HYG-F | CGGCATCTACTCTATTCCTTTGCCCTCG |
| HY-R | TTGCAAGACCTGCCTGAAACCG |
| YG-F | GGATGCCTCCGCTCGAAGTA |
| HYG-R | TCTAGAGTCGACAGAAGATGATATTGAAGGAGC |
| CHmgrF | CGGGGTACCACCAGGTCCAGGCGAACATTTA |
| CHmgrR | CGGGGTACCCAGAAGGAGGATTGGATGAGATAGG |
| HphSP | TTCTGCGGGCGATTTGTG |
| HphAP | AGCGTCTCCGACCTGATG |
| RFPF | GGGGTACCATGGTGAGCAAGGGCGAGGA |
| RFPR | CTTGTACAGCTCGTCCATGCCG |
| RFPtR | GGGGTACCTTACTTGTACAGCTCGTCCATGC |
| CmHmgrF | CGGCATGGACGAGCTGTACAAGATGGGCGCCCCAACCCAG |
| CmHmgrR | GGGGTACCTCATAGCTTCTCCTTGAGTGAGACAG |
| Actin-F | ATTGGTATGGGTCAGAAG |
| Actin-R | CTCGTTGTAAAAGGTGTG |
| CmHmgr1-F | GCTTCTTACTTCCTCTTC |
| CmHmgr1-R | CTGACTTGAACTGTATGC |
| CmYca1-F | TCATCACTGCTCTGAAGAA |
| CmYca1-R | ACTTAATTGCGGCTTCTG |
| CmAif1-F | CACTACGCTTTGAAACAC |
| CmAif1-R | CGGCAATTCTCCAATAGA |
| CmNdi1-F | TACTCTGACCTACCTCTT |
| CmNdi1-R | GAACACCTTGGACTTGAC |
| CmNma111-F | GATTGCGGATAATGAGTA |
| CmNma111-R | AATAGTCGGGAAGTAATG |
| CmBix1-F | TTTATCCGCAAGGTGTATG |
| CmBix1-R | CTCTTTGATGAACTTCTGGTA |
