# Supplementary figures and images for: Disruption of CmHmgr1 triggers apoptosis and causes defects in growth, conidiogenesis, and mycoparasitism of Coniothyrium minitans
Source: Virulence. 2025 Jul 23;16(1):2523884. doi: 10.1080/21505594.2025.2523884 (PMC12296070; doi:10.1080/21505594.2025.2523884)

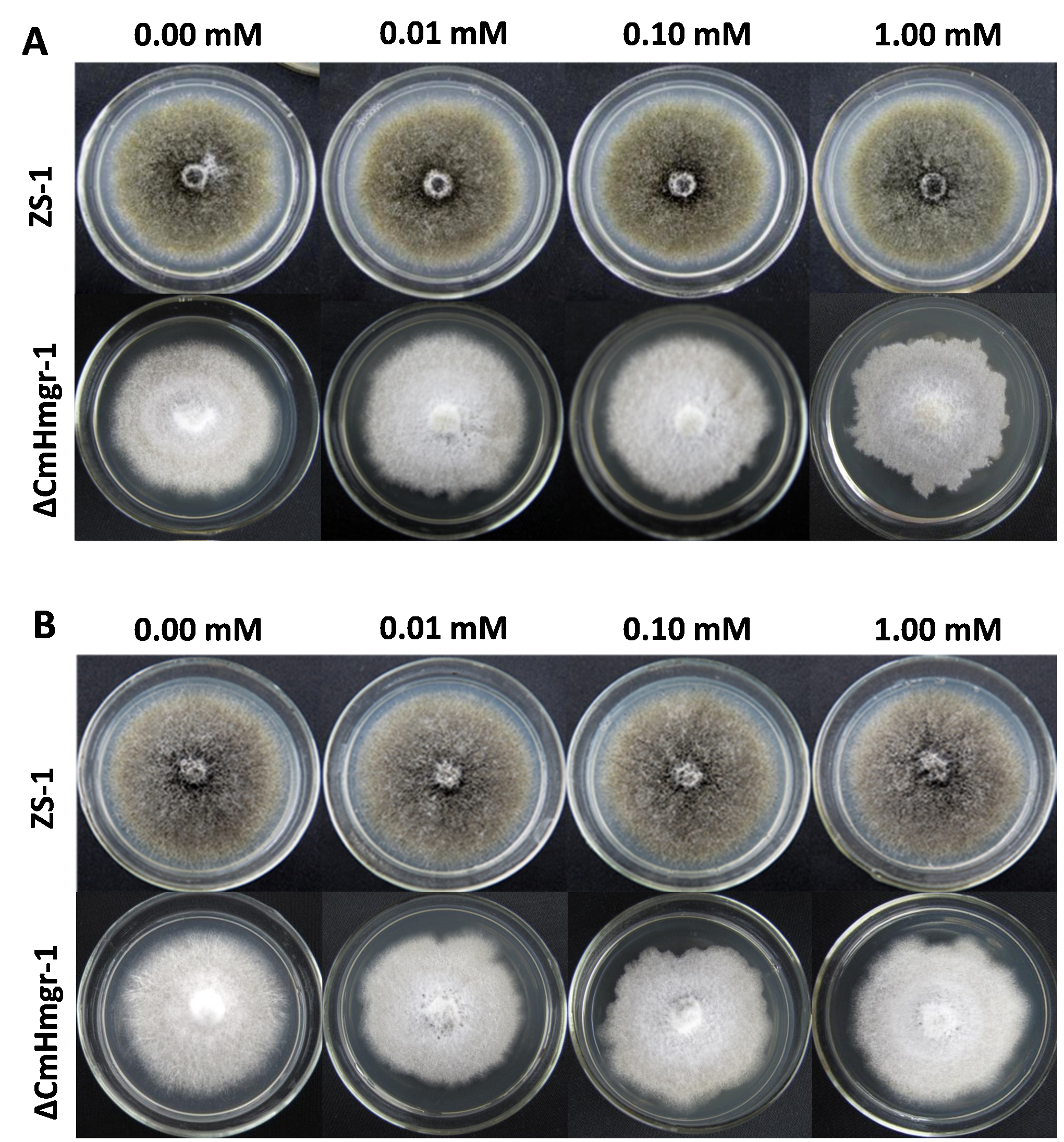

Supplement: Figure S1.tif [file KVIR_A_2523884_SM9338.tif]

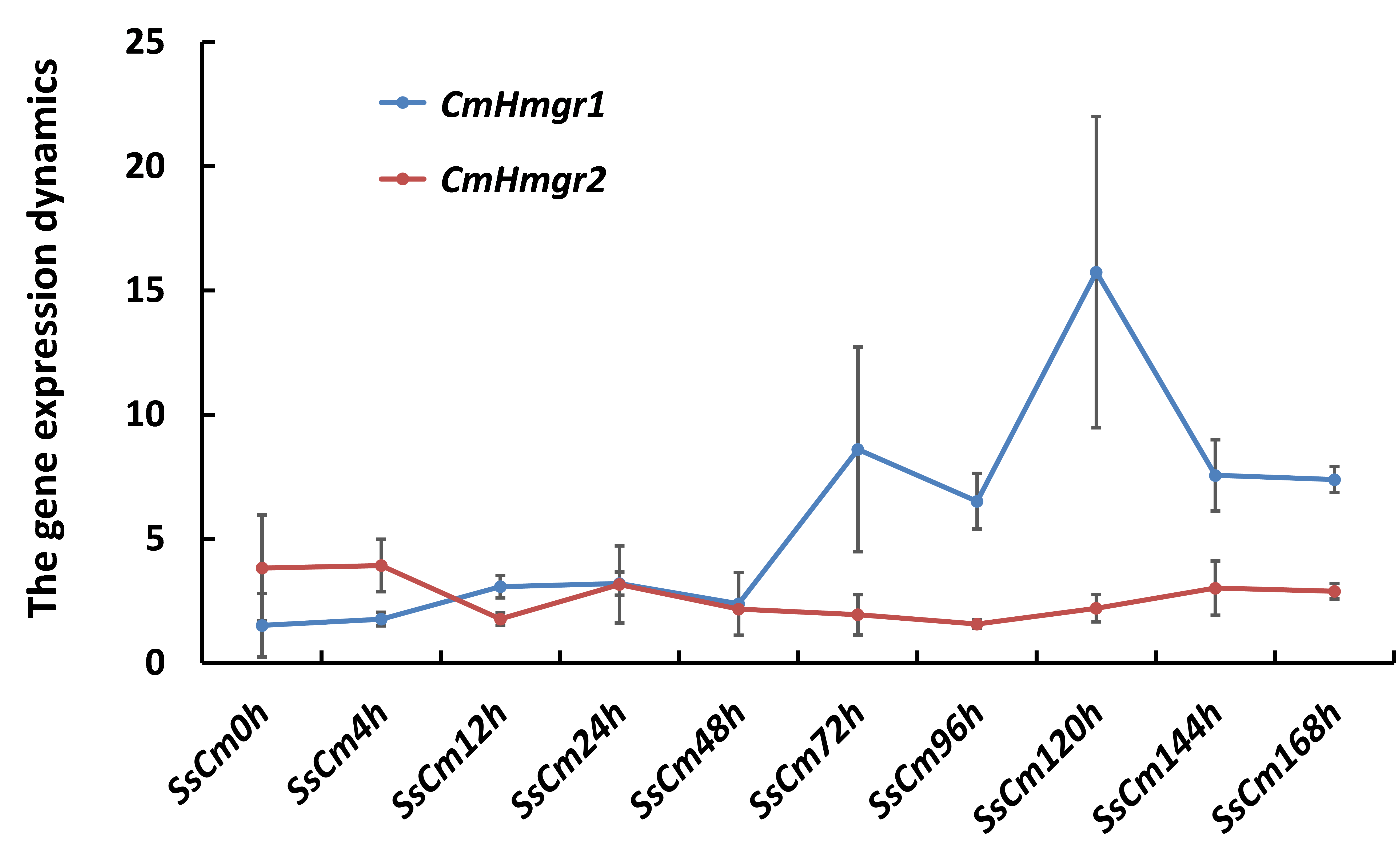

Supplement: Figure S2.tif [file KVIR_A_2523884_SM9337.tif]
